# Cerebral aspergillosis in a patient with leprosy and diabetes: a case report

**DOI:** 10.1186/1756-0500-7-689

**Published:** 2014-10-04

**Authors:** João Batista Alves Segundo, Marcos Antonio Custódio Neto da Silva, Walbert Edson Muniz Filho, Anna Cyntia Brandão Nascimento, Flávia Castello Branco Vidal, Geusa Felipa de Barros Bezerra, Graça Maria de Castro Viana, Maria do Desterro Soares Brandão Nascimento

**Affiliations:** Medicine Course, Federal University of Maranhão, Gonçalves Dias Square, s/n, São Luís, Maranhão Brazil; Department of Pathology, Nucleum of Basic and Applied Immunology, Federal University of Maranhão, São Luís, Maranhão Brazil; University Hospital of Federal University of Maranhão, Street Barão of Itapary, 227, Center, São Luís, Maranhão Brazil; Department of Morphology, Federal University of Maranhão, São Luís, Maranhão Brazil; Postgraduation Program in Maternal-Child Health, Nucleum of Basic and Applied Immunology, Portugueses Avenue, 1966, Bacanga. Prédio do CCBS, Bloco 3, Sala 3ª, São Luís, MA CEP 65080-805 Brazil; Medicine Course, State University of Maranhão, Caxias, Maranhão Brazil

**Keywords:** Cerebral aspergillosis, Leprosy, Diabetes, Mycotic arteritis, *Aspergillus fumigatus*

## Abstract

**Background:**

Opportunistic fungi are dispersed as airborne, ground and decaying matter. The second most frequent extra-pulmonary disease by *Aspergillus* is in the central nervous system.

**Case presentation:**

The case subject was 55 years old, male, mulatto, and an assistant surveyor residing in Teresina, Piauí. He presented with headache, seizures, confusion, fever and left hemiparesis upon hospitalization in 2006 at Hospital São Marcos. Five years previously, he was diagnosed with diabetes mellitus, and 17 months previously he had acne margined by hyperpigmented areas and was diagnosed with leprosy. Laboratory tests indicated leukocytosis and magnetic resonance imaging showed an infarction in the right cerebral hemisphere. Cerebrospinal fluid examination showed 120 cells/mm^3^ and was alcohol-resistant bacilli negative. Trans-sphenoidal surgery with biopsy showed inflammation was caused by infection with *Aspergillus fumigatus*. We initiated use of parenteral amphotericin B, but his condition worsened. He underwent another surgery to implant a reservoir of Ommaya–Hickmann, a subcutaneous catheter. We started liposomal amphotericin B 5 mg/kg in the reservoir on alternate days. He was discharged with a prescription of tegretol and fluconazole.

**Conclusion:**

This report has scientific interest because of the occurrence of angioinvasive cerebral aspergillosis in a diabetic patient, which is rarely reported. In conclusion, we suggest a definitive diagnosis of cerebral aspergillosis should not postpone quick effective treatment.

## Background

Opportunistic fungi are dispersed in nature as airborne particles from soil and mulch [1, 2] and infection results from aspiration of conidia in the air, especially in humid environments [3]. *Aspergillus fumigatus*, *Aspergillus flavus* and *Aspergillus niger* species account for 95% of infections in humans [4]. *Aspergillus* infection becomes more important in immunocompromised patients, such as transplanted patients, human immunodeficiency virus carriers and patients undergoing cancer treatment [5]. The most common type of infection is invasive pulmonary aspergillosis (80–90%) that can spread to the central nervous system (CNS) in 10–25% of cases. The second most common extra-pulmonary disease is that of the CNS [6].

The fungus can reach the brain through the blood by contiguity through the cribriform walls of the sphenoid sinus and cavernous sinus, optic nerve or vascular walls, or by direct implantation through neurosurgery [3]. The most common characteristic clinical symptoms of infection are headache, altered mental status and seizures [7]. Patients may manifest seizures or focal neurological signs from mass effect or stroke [8]. Diagnostics are performed by imaging and fungus can be measured in cerebrospinal fluid (CSF) using Sabouraud agar with culture medium [4, 9]. Surgical treatment should be early and aggressive with the purpose of eliminating most of the necrotic material via sinus surgery or craniotomy [10].

## Case presentation

This paper presents a case report of invasive aspergillosis of the CNS with mycotic carotid arteritis. Early diagnosis and appropriate treatment are essential for a good prognosis.

The case subject was a 55-year-old male, mulatto, who was an assistant surveyor residing in Teresina (PI). Five years previously, he was diagnosed with diabetes mellitus and started on treatment with neutral protamine hagedorn (NPH) insulin + metformin. During this period, the patient had one episode of decreased level of consciousness.

Seventeen months previously, he presented with margined hyperpigmented dermatosis and was diagnosed with leprosy. He started treatment with dapsone for 14 months. He reported headaches six months ago, initially related to sinusitis treated by an otolaryngologist, but then started having seizures and was transferred to a neurologist who initiated anticonvulsants. The patient was not involved in gardening or agricultural activities and he was not a smoker. The patient worked as an assistant surveyor in the measurement of land, which could cause potential exposure to fungi from the air and soil.

Three months previously, he developed mental confusion, fever and left hemiparesis, and was admitted to São Marcos Hospital on 2 March 2006. He was prescribed NPH insulin, metformin 850 mg, rocefin 2 g/day, meticorten, tegretol 600 mg/day and gardenal 100 mg/day.

**Figure 1:**
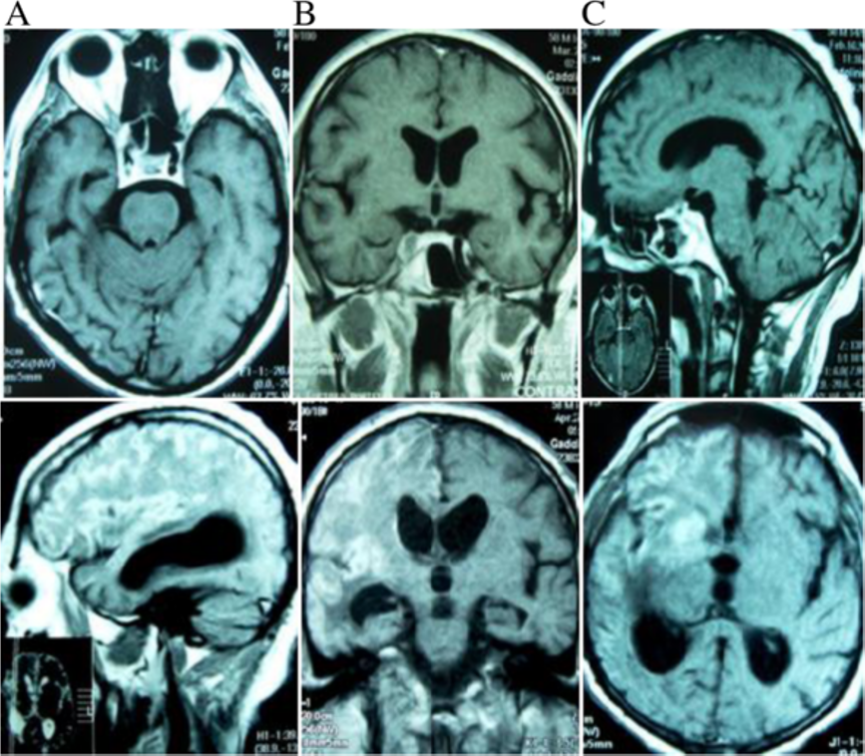
**Magnetic Resonance Imaging in T1-weighted series of (A) axial, (B) coronal, and (C) sagittal sections.** The hyperintense areas in topography of the right sphenoid sinus are evident in the three images, suggestive of fungal sinusitis. Hyperintense areas in the right superolateral cortical regions are evident in the three images, suggestive of cerebral infarction with luxury hyperemia by arteritis.

Laboratory and imaging tests were conducted. The hemogram on 2 March 2006 showed leukocytosis was 10.300 cells/mm^3^, 0.7 mg% creatinine and glucose 160 mg%. Chest X-ray showed pleural thickening with obliteration of the left costophrenic sinus on 4 March 2006. Computed tomography (CT) and magnetic resonance imaging (MRI) on 9 March 2006 showed right cerebral hemisphere infarction with hyperemia luxury, thrombosis of the carotid artery and sphenoid expansive process with cavernous sinus invasion, meningeal base and hydrocephalus (Figure 1). Lumbar puncture was performed with CSF examination (14 March 2006), which showed 120 cells/mm^3^, 69% lymphocytes, 49 mg% protein and 87 mg% glucose with negative alcohol resistant bacilli (BAAR) in the CSF.

**Figure 2:**
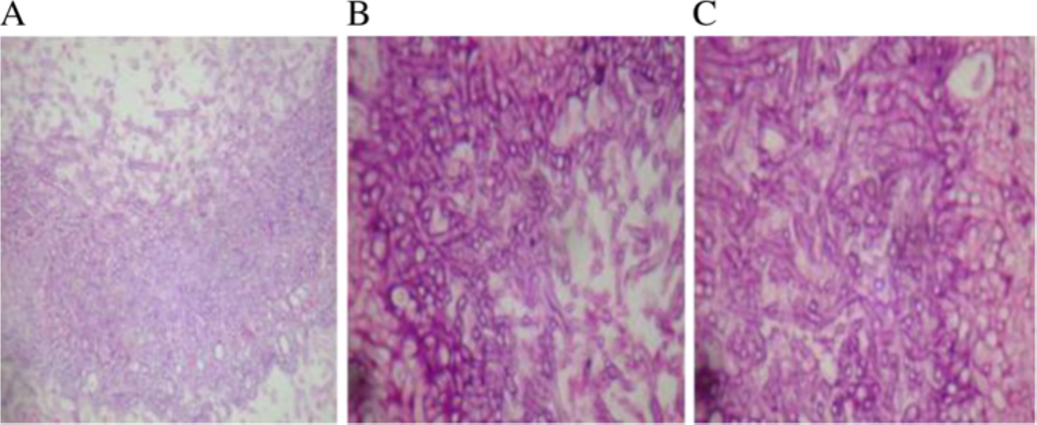
**Histopathological sections obtained by sphenoid biopsy demonstrating the presence of septate dichotomous hyphae by microscopy, suggesting** ***Aspergillus spp*** **.** Hematoxylin Eosin **(A)** magnification is 40× and **(B and C)** 400× .

After examination, the patient was transferred from the clinical neurologist to a neurosurgeon, otolaryngologist and infectologist. He underwent trans-sphenoidal surgery with biopsy on 4 March 2006, which showed inflammation and intense infection by *Aspergillus fumigatus* by hematoxylin-eosin staining of biopsy samples (Figure 2).

Parenteral liposomal amphotericin B (5 mg/kg/day) treatment was initiated but there was worsening of symptoms with a decreased level of consciousness after intensification of convulsive seizures and vomiting. Liposomal amphotericin B is an alternative for the treatment of choice in some cases. Here, liposomal amphotericin B was initiated because of its lower cost compared to voriconazole.

A second surgery was performed on 17 May 2006 to implant a subcutaneous Ommaya - Hickmann reservoir and intra-ventricular catheter. Ventricular CSF was collected and examination showed 820 cells/mm^3^, 58% neutrophils, 106 mg% protein and 31 mg% glucose, with negative culture.

A new cranial CT scan showed right cerebral hemisphere infarction, and a catheter was placed into the right lateral ventricle and there was a reduction of hydrocephalus. Liposomal amphotericin B 5 mg/kg by reservoir on alternate days was initiated while maintaining NPH parenteral insulin and tegretol l.600 mg/day. The Infectious Diseases Society (IDSA) guidelines recommend 3–5 mg/kg/day of liposomal amphotericin B for treatment of cerebral aspergillosis. The scheme was administered on alternate days because of the patient’s clinical condition and comorbidities.

The galactomannan test was not performed. Diagnosis of fungal infection was made by histopathology. Histopathological sections obtained by sphenoid biopsy demonstrated the presence of septate hyphae with dichotomous branching, suggesting *Aspergillus* spp.

There was progressive clinical improvement, seizures stopped, and the patient awoke, could feed orally and walk, with support, with reduced left hemiparesis. The patient was discharged on 14 June 2006 with a prescription of 600 mg/day tegretol + 150 mg fluconazol 2 capsules/day.

## Discussion

*Aspergillus* dissemination to the CNS is a devastating complication of invasive aspergillosis [11–13]. CNS aspergillosis is the most lethal manifestation of *Aspergillus* infection with a mortality rate of > 90% [12].

*Aspergillus* infection often occurs in patients with weakened immune systems, such as transplant patients, HIV carriers and patients undergoing cancer treatment [14]. Other factors of immunosuppression include diabetes mellitus and leprosy, comorbidities previously shown by case reports [15]. Although people have contact with a variety of species of *Aspergillus*, only seven species are implicated in human infections. *Aspergillus fumigatus* is responsible for about 90% of infections, followed by *Aspergillus flavus* [16].

The main route of contamination of the CNS is hematogenous dissemination and contiguity from an adjacent area, such as the orbit or paranasal sinuses [10, 17, 18]. The hyphae may block intracerebral blood vessels, promote infarction that is commonly hemorrhagic and sterile, and can progress to a septic abscess [9, 14, 19–22] that promotes mixed inflammation reactions, necrosis [21, 22], vasculitis and mycotic aneurysms [9, 20, 22].

There have been few reports of invasive cerebral aspergillosis in patients with diabetes [23], indicating the relevance of this study as the patient had diabetes mellitus and leprosy as factors impairing the immune system. Oddo and Acuña [24], in a study of 5,612 necropsies, found 175 cases of opportunistic infection, and aspergillosis totaled 41 cases (23.4%) ranking second, behind candidiasis.

Clinical manifestations result from the fungus pathogenicity and the host immune response [25]. The presence of seizures confirms the case reported by Nogales-Gaete *et al.* [3]. The diagnosis of aspergillosis is difficult and complex because of its nonspecific signs and symptoms [26], and is performed by imaging tests that show changes that must then be correlated with clinical, histopathological and laboratory tests (culture and serology).

Early diagnosis is very important for the management of mold infections of the CNS to allow for timely therapeutic intervention and prevention of neurologic sequelae. CT and MRI are important adjuncts in the detection of infection and in monitoring the course of therapy [27].

Detection of galactomannan antigen and 1,3-β-dglucan in CSF can assist in the diagnosis of cerebral aspergillosis and other mold infections [28, 29]. However, as these antigens can also be produced by other species of fungi such as *Fusarium*, *Scedosporium* and *Exserohilum rostratum*, the detection of these antigens do not provide definitive diagnosis of cerebral aspergillosis [30]. A polymerase-chain-reaction assay specific for aspergillus might be useful, but standardized platforms are lacking [31].

There have been few randomized trials on the treatment of invasive aspergillosis. Most observations of treatment of CNS aspergillosis are based on open-label studies. One randomized trial for invasive aspergillosis demonstrated a trend toward improvement of CNS aspergillosis in patients treated with voriconazole [32].

Itraconazole, posaconazole, or liposomal amphotericin B are recommended for patients who are intolerant or refractory to voriconazole. Among the amphotericins, liposomal amphotericin B showed favorable responses in some case reports [33–35], in agreement with the current study.

Nabika *et al.* [36] reported that surgical reduction of aspergilloma combined with local administration of antifungal was a good treatment option, corroborating the present study. According to Pianetti *et al.* [25], fungal infections observed as a mass should be treated by aggressive surgical resection. A patient with recurrence may benefit from the application of intralesional amphotericin B [37]. There have been few descriptions of patients with cerebral aspergillosis that survived after undergoing surgery and antifungal therapy [20, 22].

## Conclusion

This report has scientific interest because of the occurrence of angioinvasive cerebral aspergillosis in a diabetic patient, findings that are rare in the literature. In conclusion, a definitive diagnosis of cerebral aspergillosis should not postpone treatment.

## Consent

This study was approved by the Ethics and Research of the University Hospital of the Federal University of Maranhão (233/09). The Statement of Informed Consent Form was presented to the patient and signed in accordance with Resolution No. 196/96. Written informed consent was obtained from the patient for publication of this Case Report and any accompanying images. A copy of the written consent is available for review by the Editor-in-Chief of this journal.

## Abbreviations

CNS: Central nervous system
CT: Computed tomography
MRI: Magnetic resonance imaging
CSF: Cerebrospinal fluid
NPH: Neutral protamine hagedorn
BAAR: Alcohol resistant bacilli
HIV: Human immunodeficiency virus
IDSA: Infectious diseases society.
